# Intermediate monocytes in ANCA vasculitis: increased surface expression of ANCA autoantigens and IL-1β secretion in response to anti-MPO antibodies

**DOI:** 10.1038/srep11888

**Published:** 2015-07-07

**Authors:** Eóin C. O’Brien, Wayel H. Abdulahad, Abraham Rutgers, Minke G. Huitema, Vincent P. O’Reilly, Alice M. Coughlan, Mark Harrington, Peter Heeringa, Mark A. Little, Fionnuala B. Hickey

**Affiliations:** 1 Trinity Health Kidney Centre, Department of Clinical Medicine, Trinity College Dublin, St. James’ Hospital Campus, Dublin 8, Ireland; 2 Department of Rheumatology and Clinical Immunology, University of Groningen, University Medical Center Groningen, Groningen, Netherlands; 3 Department of Pathology and Medical Biology, University of Groningen, University Medical Center Groningen, Groningen, Netherlands

## Abstract

ANCA vasculitis encompasses several autoimmune conditions characterised by destruction of small vessels, inflammation of the respiratory tract and glomerulonephritis. Most patients harbour autoantibodies to myeloperoxidase (MPO) or proteinase 3 (PR3). Clinical and experimental data suggest that pathogenesis is driven by ANCA-mediated activation of neutrophils and monocytes. We investigated a potential role for distinct monocyte subsets. We found that the relative proportion of intermediate monocytes is increased in patients versus control individuals, and both MPO and PR3 are preferentially expressed on these cells. We demonstrate that MPO and PR3 are expressed independently of each other on monocytes and that PR3 is not associated with CD177. MPO expression correlates with that of Fc receptor CD16 on intermediate monocytes. Monocyte subsets respond differently to antibodies directed against MPO and PR3, with anti-MPO but not anti-PR3 leading to increased IL-1β, IL-6 and IL-8 production. In concordance with the observed higher surface expression of MPO on intermediate monocytes, this subset produces the highest quantity of IL-1β in response to anti-MPO stimulation. These data suggest that monocytes, specifically, the intermediate subset, may play a role in ANCA vasculitis, and also indicate that substantial differences exist between the effect of anti-MPO and anti-PR3 antibodies on these cells.

Anti-neutrophil cytoplasmic antibody (ANCA)-associated vasculitis (AAV) refers to a group of severe multi system autoimmune diseases affecting the microvasculature^1^. This encompasses microscopic polyangiitis (MPA), granulomatosis with polyangiitis (GPA, formally known as Wegner’s granulomatosis) and eosinophilic granulomatosis with polyangiitis (EGPA, formerly known as Churg-Strauss syndrome). In most cases AAV is characterised by autoantibodies to myeloperoxidase (MPO) or proteinase-3 (PR3)^2^,^3^. These proteins are primarily found in the cytoplasmic granules of neutrophils and lysosomes of monocytes. Substantial clinical and experimental evidence indicates that these autoantibodies drive pathogenesis of the disease^4^,^5^. GPA, EGPA and MPA share the same pathology of necrotising vasculitis of small vessels, the primary difference between them being the development of granuloma in EGPA and GPA but not MPA, with marked eosinophilia and asthma in EGPA. The majority of AAV research to date has focused on the neutrophil as the dominant cell driving pathology, with the role of the monocyte being less well examined. However, similar to neutrophils, monocytes also express the antigenic targets MPO and PR3 and macrophages are frequently found in the vascular infiltrates of both kidneys and lungs of patients with AAV^6^. In addition, ANCA have been shown to induce the production of oxygen radicals^7^ and interleukin (IL)-8^8^ in monocytes.

For many years monocytes were classified into 2 subsets based on their expression of the Fc gamma III receptor, CD16 (CD16- and CD16+ monocytes). Recently, the CD16+ subset has been subdivided based on their surface expression of the lipopolysaccharide (LPS) co-receptor, CD14, resulting in 3 distinct monocyte populations (Table 1^9^): classical (CD14^high^CD16^neg/low^), intermediate (CD14^high^CD16^high^) and non-classical (CD14^low^CD16^high^). Classical monocytes comprise the largest subset and appear to have a role in proinflammatory responses as well as having antimicrobial effects. The previous roles described for CD16+ monocytes have yet to be fully attributed to either the intermediate or non-classical subtype, but intermediate monocytes appear to have a proinflammatory role while the non-classical subset have a patrolling and anti-viral function. ANCA stimulation of neutrophils has been shown to require the Fc portion of the autoantibody for full effect^10^, suggesting that ANCA may interact with CD16 on monocytes and therefore, distinct monocyte subsets may play a key role in the pathogenesis of the disease. Differences in the proportion of monocyte subsets, particularly an increase in intermediate cells, has previously been shown in a number of autoimmune diseases including rheumatoid arthritis^11^, sarcoidosis^12^, and severe asthma^13^. We therefore postulated that distinct monocyte subsets may exhibit different responses to the autoantibodies associated with ANCA vasculitis.

## Results

### The proportion of intermediate monocytes is increased in AAV patients

**Figure 1:**
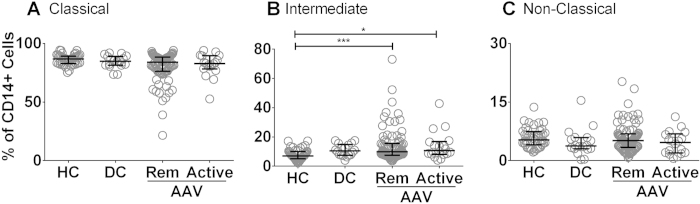
Intermediate monocyte subsets are increased in both active and remission AAV patients compared to healthy control individuals. Peripheral blood was collected from healthy control individuals, AAV patients (both patients with active disease and those in remission), and patients with other renal disease (disease controls). Percentages of monocytes in each subset (**A**–**C**) were established by flow cytometry based on CD14 and CD16 staining. Each symbol represents a separate individual. Data are presented as the median and interquartile range. (*p < 0.05, ***p < 0.005). HC: healthy control; DC: disease control; Rem: remission.

Monocyte subsets were analysed in AAV patients (n = 100, 19 active and 81 remission), disease control patients (n = 18) and healthy control individuals (n = 44) (Table 2). Individual subsets were identified based on cell surface expression of CD14 and CD16 as measured by flow cytometry. We observed no significant difference in the proportion of classical and non-classical monocytes, as a percentage of total monocytes, between healthy controls, disease controls and AAV patients (remission or active) (Fig. 1A,C). Intermediate monocytes were significantly increased in both remission and active AAV patients when compared to healthy control individuals (Fig. 1B). The fraction of intermediate monocytes in the disease control group was numerically similar to the vasculitis groups, but the increase was not significantly different from the healthy control group.

### MPO and PR3 antigens are preferentially expressed on intermediate monocytes

**Figure 2:**
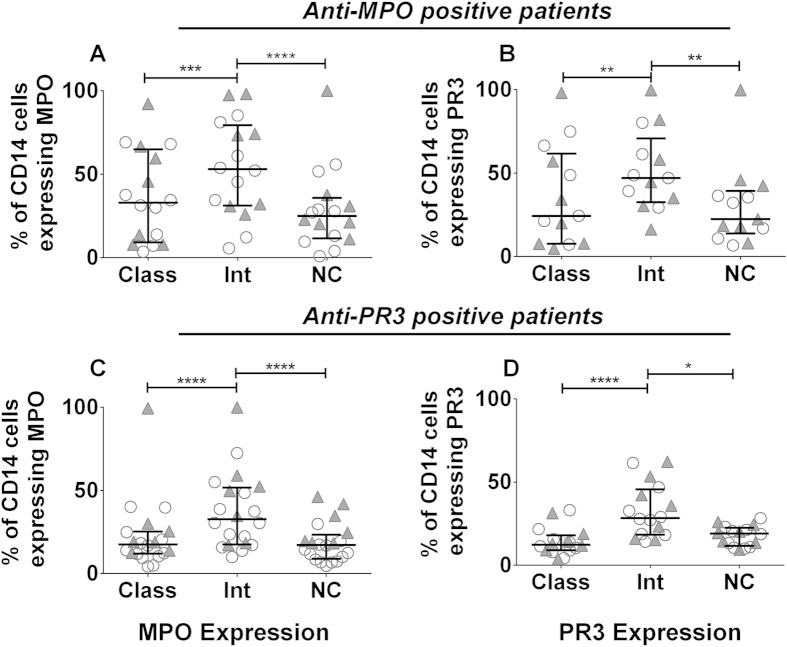
The ANCA autoantigens MPO and PR3 are preferentially expressed on intermediate monocytes. Peripheral blood was collected from patients with AAV and the percentage of cells expressing cell-surface MPO and PR3 was examined by flow cytometry. The percentage of MPO and PR3 positive cells in each subset is shown for (**A**–**B**) anti-MPO+ AAV patients and (**C**–**D**) anti-PR3+ AAV patients. Each symbol represents a separate individual. Open circles represent patients in remission and filled triangles patients with active disease. Data are presented as the median and interquartile range. Non-parametric one-way ANOVA (Friedman test) and Dunn’s post-test were used to test for significance (*p < 0.05, **p < 0.01; ****p < 0.0001). Class: classical; Int: intermediate: NC: non-classical.

We hypothesised that surface expression levels of the MPO and PR3 autoantigens may vary between the different monocyte subsets. Using a subset of the full cohort described above and following gating on each subset, the percentage of monocytes expressing cell-surface MPO was significantly increased on intermediate cells when compared to classical and non-classical subsets (Fig. 2A,C, Supplemental Fig. S1). This finding was consistent in both test and control groups, suggesting that it is a fundamental feature of the intermediate monocyte subset. Similarly, cell-surface PR3 expression was increased on the intermediate monocyte subset in both the test and control groups (Fig. 2, Supplemental Fig. S1). Following gating of MPO/PR3 positive cells in each subset we found a significant increase in the median fluorescence intensity of MPO and PR3 on intermediate monocytes (Supplemental Fig. S2).

### Monocyte surface expression of MPO and PR3 is not linked

**Figure 3:**
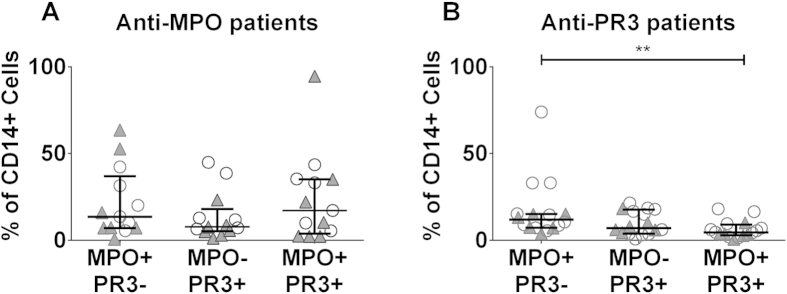
Cell-surface expression of MPO and PR3 is not linked on monocytes. Peripheral blood was collected from patients with AAV and the percentage of cells expressing surface MPO and PR3 was examined by flow cytometry. Cells were classified as being MPO+PR3-, MPO–PR3+, MPO+PR3+. Data are presented for (**A**) anti–MPO+ patients and (**B**) anti-PR3+ patients. Each symbol represents an individual patient. Open circles represent patients in remission and filled triangles patients with active disease. Data are presented as the median and interquartile range. Non-parametric one-way ANOVA (Friedman test) and Dunn’s post-test were used to test for significance (**p < 0.01).

**Figure 4:**
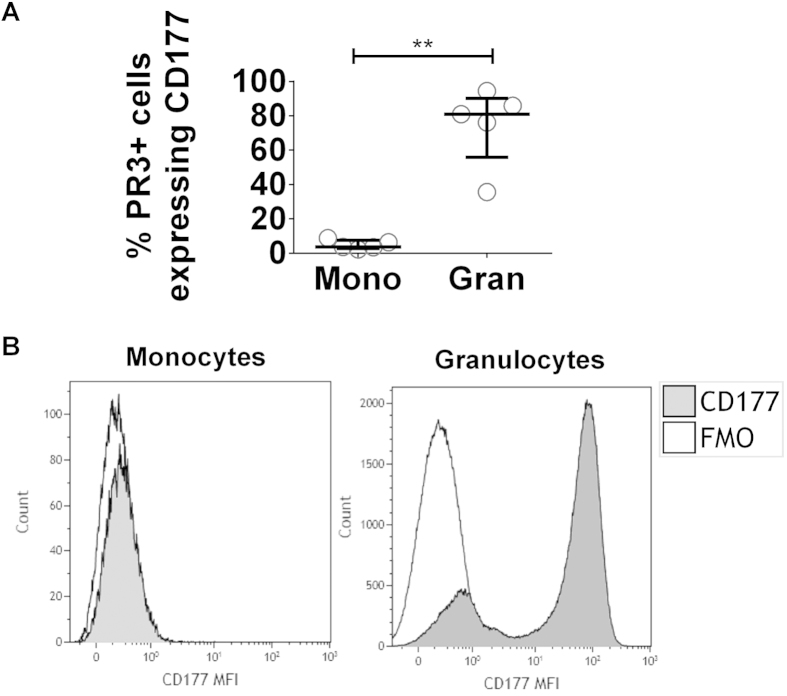
PR3+ monocytes do not co-express CD177. Peripheral blood was collected from healthy control individuals, patients with AAV and disease controls and analysed by flow cytometry. Following gating on monocytes or granulocytes the percentage of PR3+ cells in each population which co-express CD177 was determined (**A**). Representative histograms show the median fluorescence intensity (MFI) of CD177 on monocytes and granulocytes (**B**). Data represent the median and interquartile range. Paired t-test was used to test for significance (**p < 0.01). Mono: monocytes; Gran: granulocytes; FMO: fluorescence minus one control.

As cell-surface expression of both MPO and PR3 was increased on intermediate monocytes we investigated whether expression of the two autoantigens was linked in this cell type. The majority of autoantigen expressing monocytes were positive for either MPO or PR3 alone, indicating that MPO and PR3 are expressed independently on the cell surface (Fig. 3 and Supplemental Fig. S3). These data suggest that the mechanism for anchoring the antigen to the plasma membrane is different in each case. In neutrophils, surface expression of CD177 is linked to PR3 expression, with increased membrane expression of PR3 dependent on CD177^14^. We investigated whether the same mechanism accounted for surface PR3 expression on monocytes. We found little expression of CD177 on monocytes and no association between CD177 and PR3 (Fig. 4). As demonstrated previously a high proportion of PR3+ granulocytes co-expressed CD177 (Fig. 4).

### CD16 expression correlates with cell-surface MPO but not PR3 expression on intermediate monocytes

**Figure 5:**
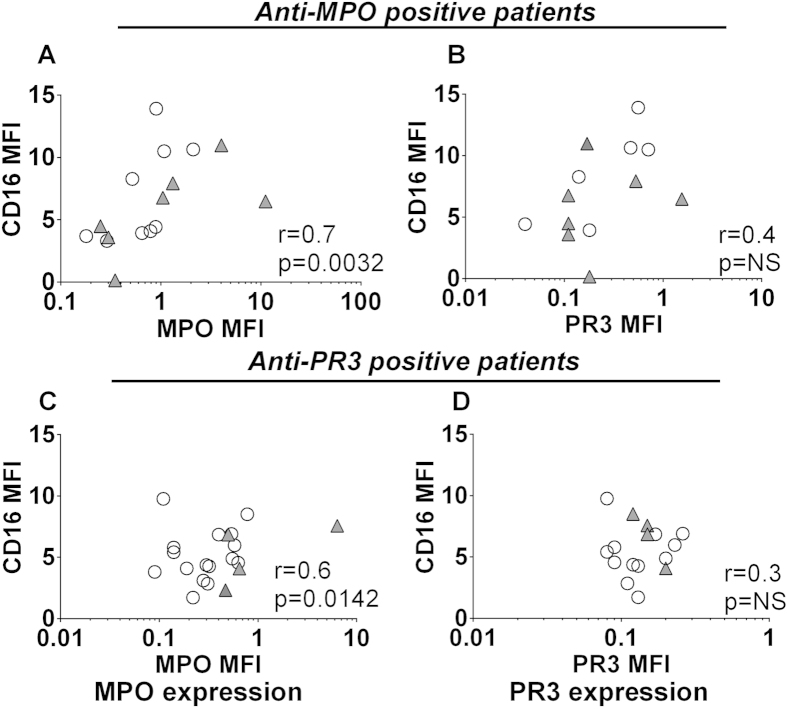
MPO and CD16 expression are correlated on intermediate monocytes. Peripheral blood was collected from patients with AAV and analysed by flow cytometry. Following gating on intermediate monocytes the MFI of CD16 was plotted against that of MPO or PR3. Data presented show (**A**–**B**) anti-MPO+ AAV patients, (**C**–**D**) anti-PR3+ AAV patients. Each symbol represents an individual patient. Open circles represent patients in remission and filled triangles show patients with active disease. Correlation was tested by Spearman Rank Test.

As CD16 positivity is the criterion for differentiation between classical and intermediate monocytes, as well as being a potential signalling mechanism for ANCA in monocytes, we investigated the relationship between CD16 and MPO/PR3. Following gating on intermediate monocytes, we assessed whether the MPO/PR3 median fluorescence intensity was correlated with that of CD16. We found that surface MPO, but not PR3, was significantly correlated with CD16 (Fig. 5). Neither antigen was correlated with CD16 in healthy controls, but it was correlated in disease controls (Supplemental Fig. S4), suggesting that this may be a feature of either renal dysfunction or systemic inflammation.

### Antibodies directed against MPO stimulate IL-1β, IL-6 and IL-8 production in monocytes

**Figure 6:**
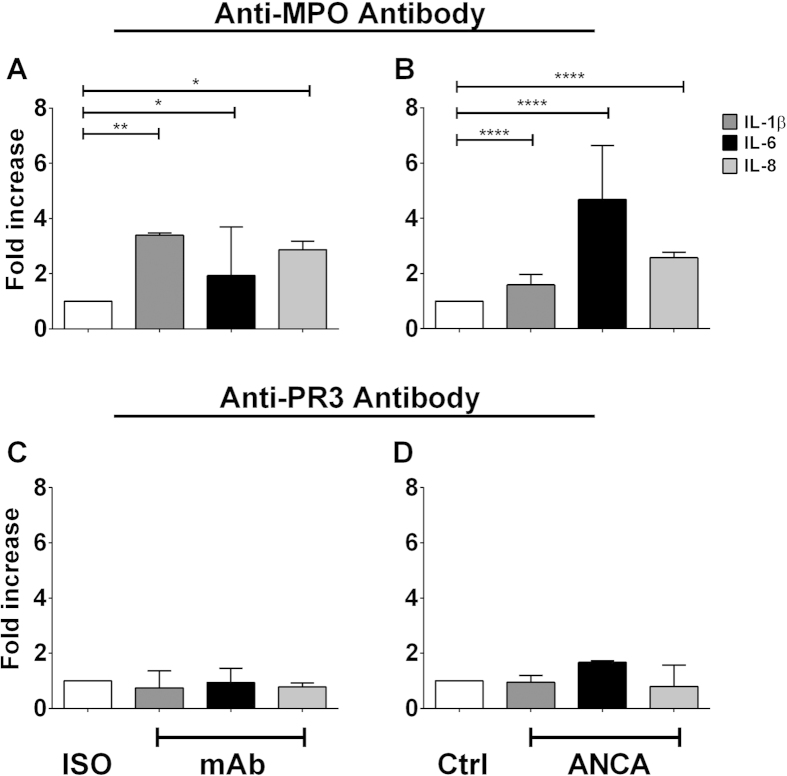
Stimulation of monocytes with anti-MPO mAb or with anti-MPO+ ANCA leads to increased IL-1β, IL-6 and IL-8 production. CD14+ monocytes were isolated from healthy controls PBMCs by MACS separation. The cells were plated and incubated with 5 ng/ml TNF-α @ 37 ^°^C for 30 minutes and then stimulated for 4 hours with 5 μg/ml of either (**A**) monoclonal antibody (mAb) directed against MPO, (**B**) protein G purified IgG from anti-MPO+ patients, (**C**) mAb against PR3 or (**D**) IgG purified from anti-PR3+ patients. Supernatants were then removed and levels of IL-1β, IL-6 and IL-8 measured by ELISA. Data are presented as the median and interquartile range of the fold increase over control. Statistical analysis was performed using Wilcoxon signed rank test (*p < 0.05, **p < 0.01, ****p < 0.0001). Iso: isotype control; CTRL: Control.

To investigate activation of monocytes following binding of ANCA to surface antigen, we measured release of pro-inflammatory cytokines IL-1β, IL-6, IL-8 and IL-12p70. Monocytes were purified from peripheral blood mononuclear cells (PBMCs) by positive selection based on CD14 expression, primed with tumour necrosis factor (TNF)-α and stimulated with either monoclonal antibodies (mAb) directed against MPO or PR3 or with immunoglobulin G (IgG) purified from the plasma of patients with AAV. Treatment with anti-MPO mAb resulted in significantly increased IL-1β, IL-6 and IL-8 production (Fig. 6A). This increase in inflammatory cytokine production was also observed when monocytes were stimulated with IgG purified from anti-MPO+ AAV patients (Fig. 6B). Interestingly, this effect was not seen in monocytes stimulated with either mAb directed against PR3 (Fig. 6C) or protein G purified IgG from anti-PR3+ AAV patients (Fig. 6D). Secretion of IL-12p70 was not detected under any of the conditions tested.

### Cytokine production in response to stimulation with anti-MPO mAb varies between monocyte subsets

**Figure 7:**
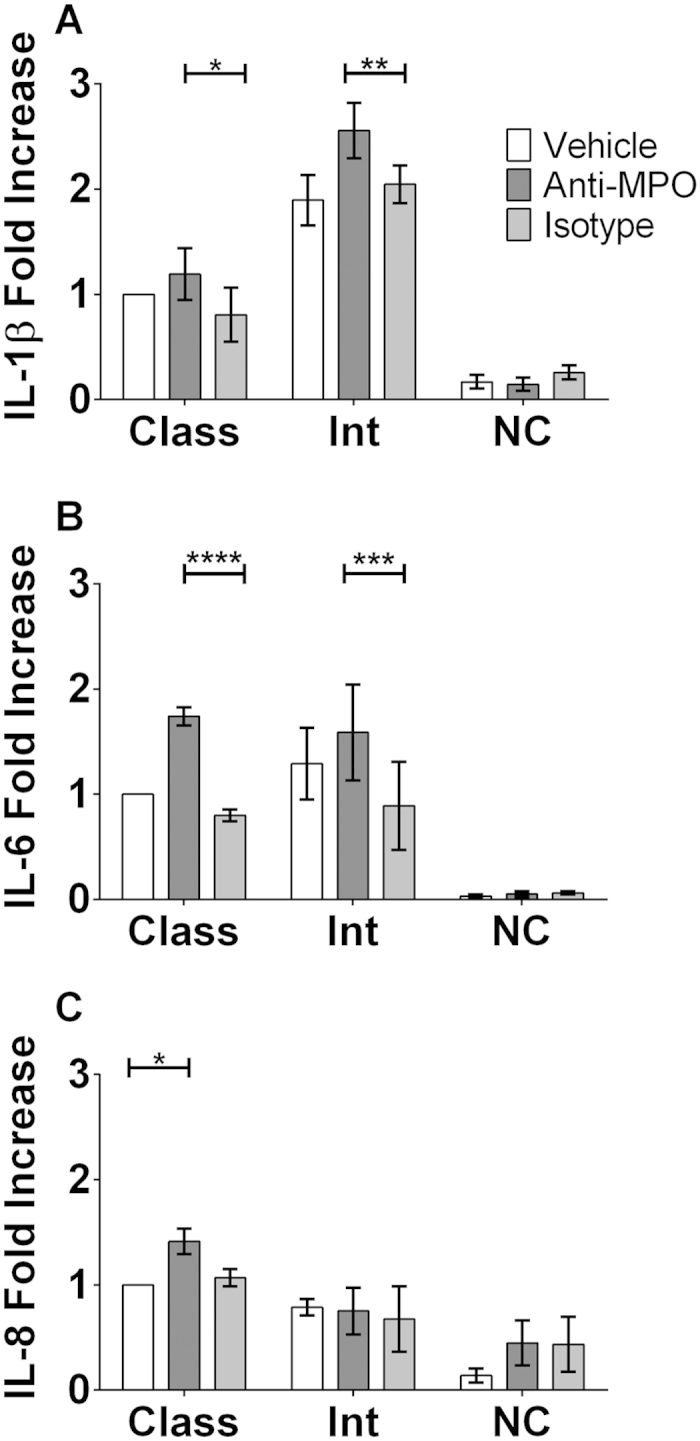
Intermediate monocytes produce increased amounts of IL-1β and IL-6 both basally and in response to stimulation with anti-MPO mAb. CD14+ monocytes were isolated from PBMCs of healthy control individuals by MACS separation. Monocyte subsets were then sorted from CD14+ cells based on CD14 and CD16 expression. Sorted subsets of cells were incubated with 5 ng/ml TNF-α @ 37 ^°^C for 30 minutes followed by stimulation for 4 hours with 5 μg/ml of either monoclonal antibody (mAb) directed against MPO or an isotype control. Supernatants were then removed and levels of (**A**) IL-1β, (**B**) IL-6 and (**C**) IL-8 measured by ELISA. Data are presented as the median and interquartile range of the fold increase over control. Statistical analysis was performed using Two-way ANOVA and Sidak test to correct for multiple comparisons (*p < 0.05, **p < 0.01, ***p < 0.001, ****p <0.0001). Class: Classical; Int: Intermediate; NC: Non-Classical

As we found MPO to be differentially expressed on different monocyte subsets, we next investigated if the production of IL-1β, IL-6 and IL-8 observed following stimulation of total monocytes differed in a subset-specific manner. Classical, intermediate and non-classical cells were sorted from MACS purified monocytes based on CD14 and CD16 expression (Supplemental Fig. S5). Sorted cells were primed with TNF-α and treated with anti-MPO mAb. This stimulation failed to induce secretion of any of the cytokines tested (IL-1β; IL-6; IL-8) from non-classical monocytes (Fig. 7A–C). IL-1β production was found to vary most between monocyte subsets, with intermediate monocytes producing higher quantities than classical monocytes both basally and in response to anti-MPO stimulation (Fig. 7A). IL-6 production was found to be similar in classical and intermediate monocytes and to be increased in both subsets following incubation of cells with anti-MPO (Fig. 7B). Conversely only classical monocytes were found to secrete IL-8 in response to anti MPO mAb (Fig. 7C).

### IL-1β production in response to anti-MPO is not dependent on Fc binding in monocytes

**Figure 8:**
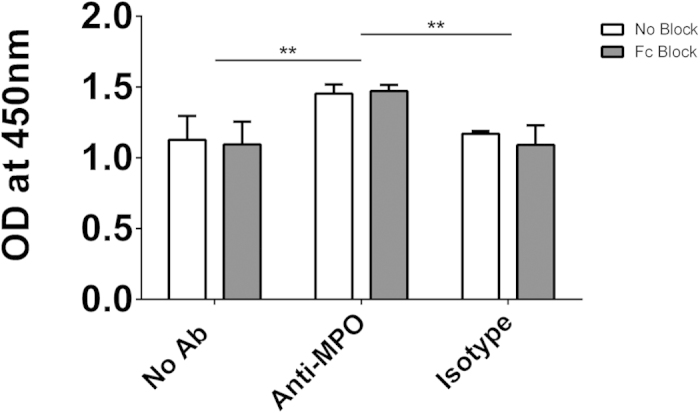
Fc receptor binding is not required for anti-MPO-induced IL-1β production by monocytes. CD14+ monocytes were isolated from PBMCs of healthy control individuals by MACS separation. Cells were incubated with 2.5 μg/1 × 10^6^ cells Fc Block @ 37 ^°^C for 30 minutes followed by stimulation for 4 hours with 5 μg/ml of either monoclonal antibody (mAb) directed against MPO or an isotype control. Supernatants were then removed and levels of IL-1β measured by ELISA. Data are presented as the median and interquartile range. Statistical analysis was performed using Two-way ANOVA and Sidak test to correct for multiple comparisons (**p < 0.01).

It has been shown previously that the binding of the Fc portion of ANCA antibodies to neutrophils is required for their activation^10^. This finding, along with our own finding that the Fc receptor (CD16) expression on monocytes correlates with expression of MPO led us to investigate whether blocking the Fc receptor would abrogate the effect of anti-MPO stimulation in monocytes. In order to test this hypothesis we treated cells with anti-MPO mAb after pre-treatment with either commercially available Fc blocking solution or vehicle. Monocyte activation was measured by IL-1β production. Treatment with Fc blocking solution had no effect on the ability of anti-MPO mAb to activate monocytes (Fig. 8).

## Discussion

At the time that the stimulatory effect of ANCA on neutrophils was first described, a similar phenomenon was described in monocytes^15^. ANCA were shown to stimulate oxygen radical production^7^ and to induce production of inflammatory cytokines such as IL-8 from monocytes^8^. Following these initial studies, the focus of research has been almost exclusively on neutrophils. As several autoimmune diseases, including rheumatoid arthritis, sarcoidosis and severe asthma^11^,^12^,^13^ are characterised by an expansion of intermediate monocytes, we postulated that ANCA vasculitis would also display this phenotype. We report for the first time that the proportion of intermediate monocytes found in AAV patients is increased when compared to healthy control individuals. In addition, we demonstrate that the autoantigens associated with ANCA vasculitis (MPO and PR3) are differentially expressed on distinct monocyte subsets, with the highest expression being seen on intermediate cells. In concordance with cell surface expression of MPO on monocytes we confirm that stimulation with anti-MPO antibodies results in monocyte activation, as measured by IL-1β, IL-6 and IL-8 production, although anti-PR3 antibodies did not have this effect. Importantly, we also demonstrate that monocyte subsets respond differently to anti-MPO antibodies, with intermediate monocytes producing the highest amount of IL-1β and increased IL-6 following stimulation.

We first divided the AAV patients into those with active disease and those who were in remission based on the hypothesis that those patients with active disease would have increased inflammatory monocytes. We found that both remission and active patients had an increased proportion of intermediate monocytes when compared to the proportions observed in healthy control individuals, This indicates that this cell type is expanded in patients and therefore may play a role in disease pathogenesis or pathophysiology.

We also analysed the expression of the autoantigens targeted by ANCA on monocyte subsets. It has been reported that monocytes express both MPO and PR3 on their cell surface^16^,^17^ although their expression on different monocyte subsets has not been reported. We found that both of these antigens are expressed preferentially on intermediate monocytes when compared to the classical and non-classical populations. Although intermediate cells account for a relatively small fraction of total monocytes in peripheral blood (6–11% in healthy individuals), the proportion of this monocyte subset is increased in ANCA patients. This increase, in combination with increased expression of the autoantigens, suggests that this subset may be the monocyte target of ANCA. Not only do an increased proportion of intermediate monocytes express these antigens, but these cells express a greater amount of MPO or PR3 on their surface than their classical or non-classical equivalents. Taken together these data suggest that the intermediate subset is the monocyte population which is most susceptible to ANCA stimulation.

In anti-MPO+ AAV patients the correlation between surface MPO and CD16 on intermediate monocytes indicates that expression of these two molecules may be linked. In neutrophils, ANCA have been shown to induce the activation of the cell through first binding to MPO or PR3 and subsequent signalling through their Fc region^10^. As CD16 is an Fc receptor its expression on the same cells as MPO may provide an insight into the possible activation of these cells by anti-MPO ANCA. The differential nature of surface MPO and PR3 expression is highlighted by the fact that in AAV patients CD16 is only correlated with MPO but not PR3. The significance of this finding remains to be determined as we have found that Fc receptor binding is not required for the induction of IL-1β by anti-MPO. It has been shown that CD16 expression occurs in lipid rafts^18^. While CD16 is not directly required for signalling in response to anti-MPO stimulation, the correlation between MPO and CD16 may indicate that lipid raft formation is occurring and therefore other glycosylphosphatidylinositol (GPI)-linked proteins may be involved in the response to anti-MPO antibodies. Another way in which monocytes appear to differ from neutrophils is the way in which they express PR3 on their surface. In neutrophils, surface translocation of PR3 usually occurs through an association with CD177^14^. This does not appear to be the mechanism in monocytes; elucidation of how both PR3 and MPO traffic and remain on the plasma membrane of monocytes, as well as presumably facilitating an outward-in signalling process, will need to be examined closely. CD177 is thought to form a signalling complex through which neutrophils are stimulated following binding of antibodies to PR3^19^. As we have shown, monocytes lack CD177 on their surface and this may explain why stimulation with PR3 antibodies does not lead to IL-1β production in these cells.

It has been shown previously that mAbs directed against both MPO and PR3 lead to the production of IL-1β from monocytes^20^. However, our data demonstrate that only anti-MPO and not anti-PR3 stimulation leads to the activation and subsequent IL-1β production from monocytes. The discrepancy between these two results may be due to the fact that monocytes were isolated by different methods. We have used a positive magnetic bead selection method, whereas Schreiber *et. al.* used a plastic adherence method in order to purify their monocytes. It has been shown previously that adherence of monocytes to plastic can result in partial activation of the cells^21^. This activation may account for the differences in IL-1β production in response to ANCA stimulation.. We have shown a similar result when mAbs were replaced with IgG derived from anti-MPO + and anti-PR3+ patients, with only IgG from anti-MPO+ patients leading to IL-1β production, further verifying the specificity of the response. This specific anti-MPO response is also observed when we investigated other inflammatory cytokines, IL-6 and IL-8. The production of these cytokines mirrored the pattern observed in IL-1β production from the stimulated total monocyte population.

In order to investigate our hypothesis that the intermediate subset of monocytes, by virtue of their increased antigen expression, would have the greatest response to anti-MPO antibodies we used a similar system of stimulation to that used for total monocytes. For these experiments we went a step further and sorted the individual monocyte subsets based on their CD14 and CD16 expression. This allowed us to analyse the individual subsets and therefore show how each group was contributing to the response to antibody stimulation which we had seen previously. Stimulation of the intermediate subset led to the highest production of IL-1β. The magnitude of the increase in anti-MPO induced IL-1β in sorted monocytes was not as high for any of the subsets as had been observed in total monocytes. This is likely a consequence of the additional MoFlo sorting procedure, which may have partially activated the cells prior to stimulation. This monocyte subset has previously been shown to produce a number of cytokines in response to LPS^22^; however, their response to ANCA has not been studied until now. We have shown that the three monocyte subsets each have a different cytokine profile following stimulation with anti-MPO antibodies. While the intermediate monocytes showed the greatest increase in IL-1β production, the amount of IL-6 produced in these cells was comparable to that seen in the classical subset. Conversely, the IL-8 production observed in response to ANCA stimulation of monocytes was shown to be exclusively a product of the classical subset. Interestingly, secretion of each of these cytokines was unchanged in the non-classical subset in response to ANCA stimulation. Previous studies have attempted to classify the three monocyte subsets based on their differential cytokine production, which also serve to underline the complex nature of cytokine release by each subset^22^,^23^.

It is unclear whether intermediate monocytes represent a transitional cell type or whether they are a functionally distinct cell population. Our data clearly support a distinct functional role for these cells as they differ from both classical and non-classical monocytes in terms of autoantigen expression, production of the pro-inflammatory cytokine IL-1β, and response to ANCA. For all of these parameters, non-classical cells were more similar to the classical subset than they were to the intermediate, suggesting that intermediate monocytes are not a transitional population.

The production of IL-1β has been shown to play a critical role in the pathogenesis of ANCA vasculitis, with the IL-1 receptor antagonist anakinra protecting against glomerulonephritis in an anti-MPO-induced mouse model^20^. By sorting of individual monocyte subsets we show that intermediate monocytes preferentially express the autoantigens associated with AAV and that these cells produce the highest levels of IL-1β in response to anti-MPO, suggesting a possible key role for these cells in AAV. It is likely that activated monocytes, in combination with neutrophils, are responsible for initial pathologic responses to ANCA in patients. In this context, IL-1β has been shown to prime neutrophils for activation^24^, suggesting that activated monocytes may contribute to neutrophil activation. Our data demonstrating the induction of IL-1β, IL-6 and IL-8 by anti-MPO but not anti-PR3 antibodies highlights differences between the two primary autoantigens associated with AAV. It is increasingly appreciated that MPO-ANCA and PR3-ANCA vasculitis are different diseases both genetically and phenotypically^25^. The presence of anti-PR3 antibodies portends a high relapse rate^26^ and granulomatous disease affecting the upper and lower respiratory tract^27^,^28^, while the presence of anti-MPO antibodies is associated with a more “vasculitic” phenotype with a high incidence of scarring in kidney and lung at the time of diagnosis^2^,^28^. While mechanistic explanations linking the current work with these observations are beyond the scope of this manuscript, monocytes and macrophages are important in both the generation of granuloma^29^ and progression of fibrosis, so it is conceivable that the differential effect of anti-PR3 and anti-MPO antibodies on these cells is important in cellular pathogenesis. For example, the MCP1 / CCR2 chemokine axis is known to be pro-fibrotic^30^ and may be preferentially activated by anti-MPO antibodies. Further investigation to understand this differential effect and its link to clinical phenotype is ongoing.

## Methods

### Patients and control individuals

Blood samples from patients and both healthy and disease control individuals were obtained through the Rare Kidney Disease (RKD) Biobank and University Medical Center Groningen and written consent was obtained from all individuals. The RKD Biobank has full approval from the Trinity College Dublin Institutional Review Board and Ethics Committee, and approval from each of the individual hospitals from which samples were obtained (St. James’ hospital, Tallaght hospital and Beaumont hospital). Similarly, collection of samples at the University Medical Center Groningen was conducted according to local ethical guidelines and approved by the ethical committee of the University Medical Center Groningen, and in accordance with the Declaration of Helsinki. All AAV patients fulfilled the Chapel Hill Consensus Conference (CHCC) classification criteria^31^. Active vasculitis was defined as a Birmingham vasculitis activity score (BVAS) >3. Where possible, blood was collected from active patients prior to commencement of immunosuppression therapy. Four (24%) and twenty three (28%) of the active and remission patients with AAV were on no immunosuppression at the time of sampling. Antibodies against MPO and PR3 were determined by ELISA. The disease control group comprised patients with anti-glomerular basement membrane (anti-GBM) disease (n = 2), Takayasu’s arteritis (n = 1), uveitis (n = 1), isolated leukocytoclastic vasculitis (n = 1), IgA nephropathy (n = 1), lupus nephritis (n = 1), non-immune chronic kidney disease (n = 4), triple vessel coronary artery disease (n = 1), pyelonephritis (n = 1), cirrhosis (n = 1), megaloblastic anemia (n=1), end stage kidney disease due to ischemic nephropathy (n = 1), vascular dementia (n=1) and minimal change glomerulonephritis (n = 1).

### Flow cytometry

Peripheral blood was collected in ethylene diamine tetra-acetic acid (EDTA). Staining was performed in 100 μl of whole blood. The volume of each antibody used was 5 μl per 100 μl of blood. Samples were stained for 20 minutes in the dark with anti-CD14 Pacific Blue (RMO52, Beckman Coulter), anti-CD16 APC (3G8, Biolegend), anti-CD66b PerCP-Cy5.5 (G10F5, BD Biosciences), anti-PR3 FITC (W6M2, Abcam) and either anti-MPO PE (2C7, Serotec) or anti-CD177 PE (MEM-166, Molecular Probes). Red blood cells were lysed using 2 ml of BD FACS lysing solution diluted 1 in 10 in dH_2_O. The remaining cells were fixed with 500 μl of 2% paraformaldehyde. Flow cytometry was performed on a CyAn ADP analyser (Beckman Coulter). Single stain OneComp beads (eBioscience) and fluorescence minus one (FMO) controls were used to correct for spectral overlap and non-specific staining respectively. FACS analysis was performed using Kaluza v1.2 flow analysis software (Beckman Coulter).

### Monocyte subset analysis

Monocytes were gated first on size and granularity. Doublet cells were excluded by plotting FSC against FSC-area. Gating on CD66b positive cells (granulocytes) was used to exclude these cells from subsequent analyses. Monocytes were identified as CD66b negative, CD14 positive cells. Following gating on monocytes identified in this manner the percentage of each subset was calculated based on CD16 and CD14 expression (classical: CD14^high^CD16^neg/low^; intermediate: CD14^high^CD16^high^: non-classical: CD14^low^CD16^high^) (Supplemental Fig. S6). The percentage of MPO and PR3 positive cells was determined using gates set with FMO controls (Supplemental Fig. S7).

### Purification of monocytes and sorting of monocyte subsets

Peripheral blood was collected in EDTA. Peripheral blood mononuclear cells (PBMCs) were isolated by density gradient centrifugation using lymphoprep (Axis-Shield) and monocytes were purified by positive selection. PBMCs were incubated with anti-CD14 magnetic beads (Miltenyi Biotec) as per the manufacturer’s instructions and CD14+ cells were then isolated using an LS column on a QuadroMACS separator (Miltenyi Biotec). In experiments where monocytes were further sorted into distinct subsets, cells were stained by incubating with 5 μl of CD14 PE-Cy7 (M5E2, Biolegend) and CD16 APC (3G8, Biolegend) per 1 × 10^5^cells for 20 minutes. Cells were washed and monocyte subsets were sorted based on their expression of CD14 and CD16 using a MoFlo XDP analyser (Beckman Coulter) (Supplemental Fig. S5).

### Stimulation of monocytes and measurement of IL-1β, IL-6 and IL-8 secretion

Total CD14+ monocytes or individual monocyte subsets were isolated as detailed above. Cells were then plated in Roswell Parks Memorial Media (RPMI) supplemented with 10% fetal calf serum (FCS), 100 U/ml penicillin, 1 mg/ml streptomycin and 2 mM l-glutamine. Cells were seeded at a density of 5 × 10^5^ cells/well for total monocytes, or 3 × 10^5^ cells/well for sorted subsets. The cells were incubated with 5 ng/ml TNF-α @ 37 ^°^C for 30 minutes as used previously^20^ and then stimulated with 5 μg/ml of either monoclonal antibody (mAb) directed against MPO (2C7, Acris Antibodies) or PR3 (CLB-12.8, HiSS Diagnostics GmbH) or isotype control antibody (IgG1, Acris Antibodies), protein G purified IgG from anti-MPO + (n = 3) or anti-PR3 + (n = 3) patients or healthy controls (n = 4) for 4 hours. Supernatants were removed and IL-1β, IL-6 or IL-8 ELISA was performed as per the manufacturer’s instructions (R&D Systems).

### Fc blocking of stimulated monocytes

Monocytes were isolated and plated as detailed above. The cells were incubated with 2.5 μg Fc block (BD Biosciences)/1 × 10^6^ cells @ 37 ^°^C for 30 minutes and then stimulated with 5 μg/ml of either monoclonal antibody (mAb) directed against MPO (2C7, Acris Antibodies) or isotype control antibody (IgG1, Acris Antibodies) for 4 hours. Supernatants were removed and IL-1β ELISA was performed as per the manufacturer’s instructions (R&D Systems).

### Statistical analysis

All statistics and correlations were performed using GraphPad Prism 6 software and nonparametric analyses were used for non-normal data. When comparing 3 or more groups, a Kruskal-Wallis test was performed for unpaired samples and a post hoc Friedman test was used for paired samples. Multiple comparisons were corrected using Dunn’s multiple comparisons test. For comparisons between 2 groups, a two-tailed Mann Whitney test was used. Correlation was assessed using the Spearman rank test. To assess the difference in cytokine production between anti-MPO and isotype mAb across the 3 subsets 2-way ANOVA with Sidak’s post hoc multiple comparison test was used. Differences were not statistically significant (p < 0.05) unless specified.

## Additional Information

**How to cite this article**: O’Brien, E. C. *et al.* Intermediate monocytes in ANCA vasculitis: increased surface expression of ANCA autoantigens and IL-1β secretion in response to anti-MPO antibodies. *Sci. Rep.* **5**, 11888; doi: 10.1038/srep11888 (2015).

## Supplementary Material

Supplementary Information

## Figures and Tables

**Table 1:** Monocyte subset markers and phenotype (adapted from^32^).

Monocyte Subset	CD14/CD16 expression	Inflammatory role	% in peripheral blood median (IQR)
Classical	CD14^high^CD16^neg/low^	Pro-inflammatory, antimicrobial	84.3 (81.4–88.8)
Intermediate	CD14^high^CD16^high^	Pro-inflammatory	9.4 (7.4–13.7)
Non-classical	CD14^low^CD16^high^	Patrolling, Anti-viral	4.8 (2.8–7.3)

‘% in peripheral blood’ refers to the median and interquartile range (IQR) of all samples included in this study.

**Table 2:** Demographic and clinical information for patients and controls.

	Vasculitis(n = 100)	Active(n = 19)	Remission(n = 81)	Disease Controls(n = 18)	Healthy Controls(n = 44)
Age: Median (Range)	59 (50–70)	*55 (51*–*73)*	*59 (49*–*70)*	54 (17–87)	39 (22–75)
Gender	51 male 49 female	*14 male 5 female*	*37 male 44 female*	13 male 5 female	19 male 25 female
ANCA type at diagnosis	17 anti-MPO 83 anti-PR3	*7 anti-MPO 12 anti-PR3*	*10 anti-MPO 71 anti-PR3*	N/A	N/A
Proportion of patients on immunosuppression	72%	*76%*	*72%*	65%	0%
Median CRP (mg/dL, interquartile range)	3 (1–12)	*15(10*–*64)*	*2 (1*–*7)*	9 (1–34)	N/A
eGFR (ml/min, interquartile range)	63 (42–80)	*49 (35*–*87)*	*64 (42*–*80)*	64 (18–87)	N/A
Proportion eGFR < 60 ml/min	40%	*47%*	*38%*	44%	N/A

All clinical data were obtained on the same date as analysis of monocytes. CRP = C reactive protein.
