# Supplementary material for: Intermediate monocytes in ANCA vasculitis: increased surface expression of ANCA autoantigens and IL-1β secretion in response to anti-MPO antibodies
Source: Sci Rep. 2015 Jul 7;5:11888. doi: 10.1038/srep11888 (PMC4493694; doi:10.1038/srep11888)
Supplement: Supplementary Information [file srep11888-s1.pdf]

Intermediate monocytes in ANCA vasculitis: increased surface expression of ANCA autoantigens and IL-1β secretion in response to anti-MPO antibodies

Eóin C. O'Brien[1], Wayel H. Abdulahad[2], Abraham Rutgers[2], Minke G. Huitema[2], Vincent P. O'Reilly[1], Alice M. Coughlan[1], Mark Harrington[1], Peter Heeringa[3], Mark A. Little[1]*, Fionnuala B. Hickey[1]

[1]Trinity Health Kidney Centre, Department of Clinical Medicine, Trinity College Dublin, St. James' Hospital Campus, Dublin 8, Ireland.

[2]Department of Rheumatology and Clinical Immunology, University of Groningen, University Medical Center Groningen, Groningen, Netherlands

[3]Department of Pathology and Medical Biology, University of Groningen, University Medical Center Groningen, Groningen, Netherlands

***Address correspondence to:**

Mark Little, Trinity Health Kidney Centre, Room 1.07, Institute of Molecular Medicine – Phase I, St James' Hospital Campus, Dublin 8, Ireland.

Tel: +353-1-896-3236

e-mail: mlittle@tcd.ie

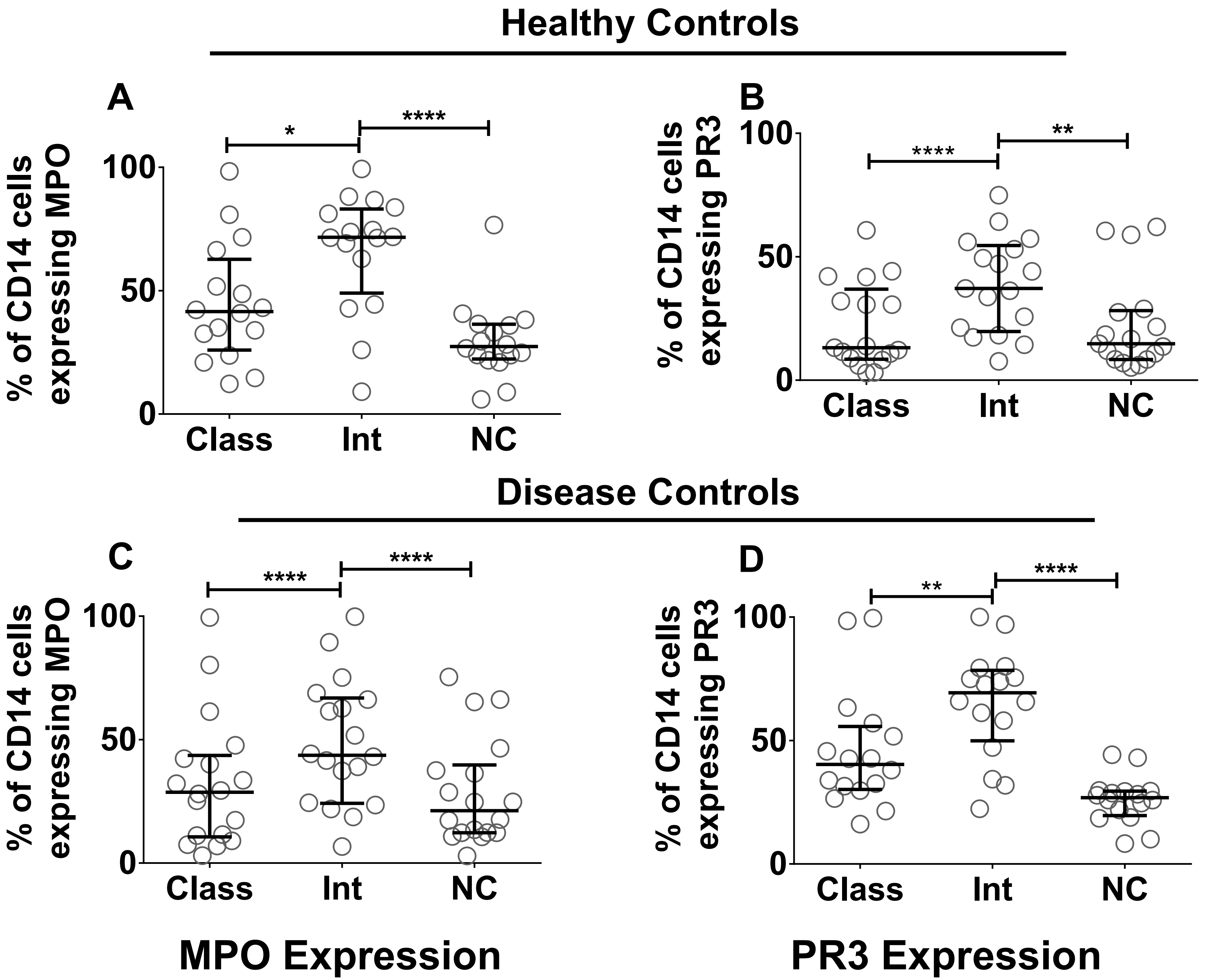
Healthy Controls
A
B
Disease Controls
C
D
% of CD14 cells expressing MPO
% of CD14 cells expressing PR3
*
**
****
0
50
100
Class
Int
NC
MPO Expression
PR3 Expression

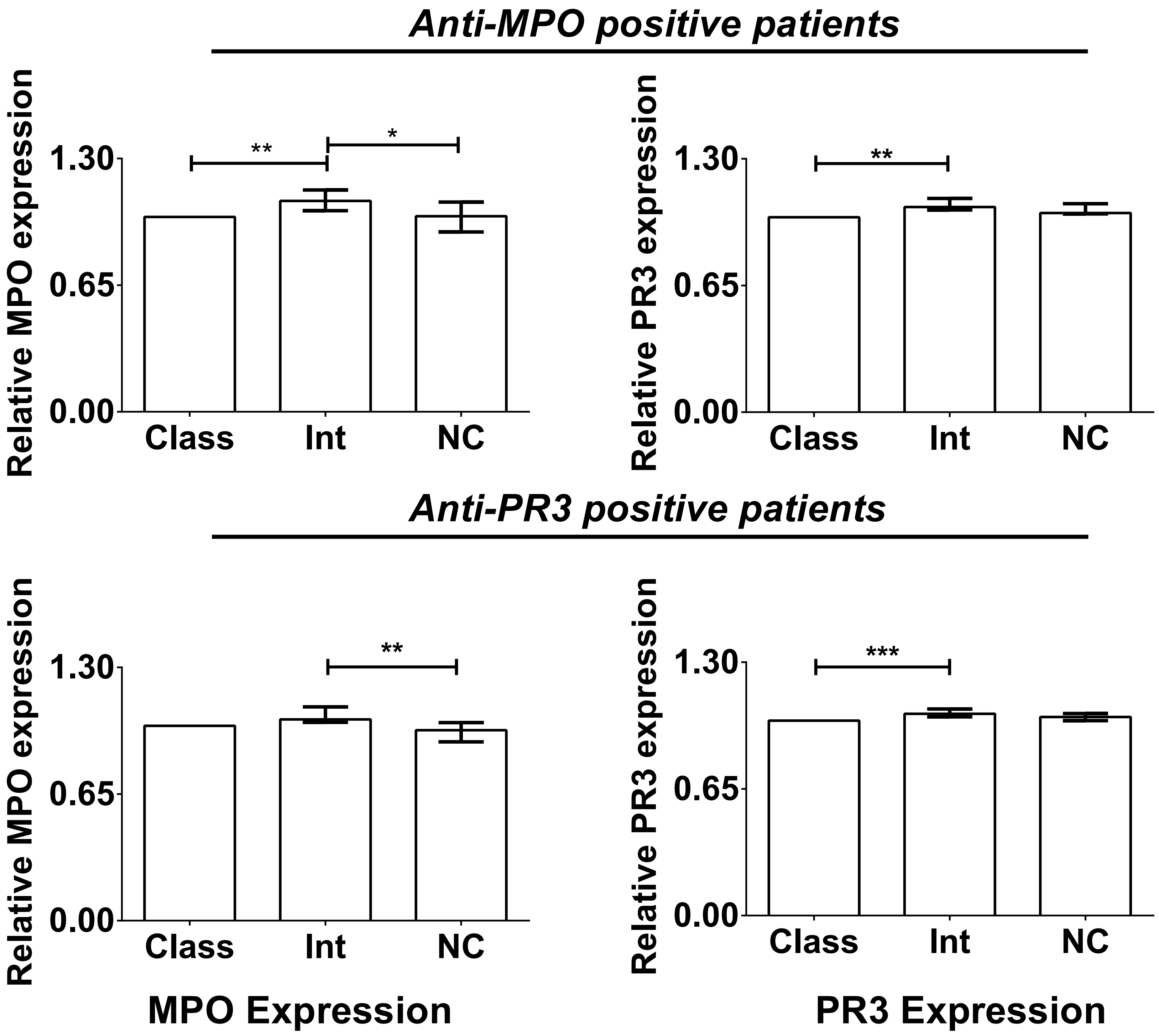
Anti-MPO positive patients
Relative MPO expression
1.30
0.65
0.00
Class
Int
NC
**
*
Relative PR3 expression
1.30
0.65
0.00
Class
Int
NC
**
Anti-PR3 positive patients
Relative MPO expression
1.30
0.65
0.00
Class
Int
NC
**
Relative PR3 expression
1.30
0.65
0.00
Class
Int
NC
***
MPO Expression
PR3 Expression

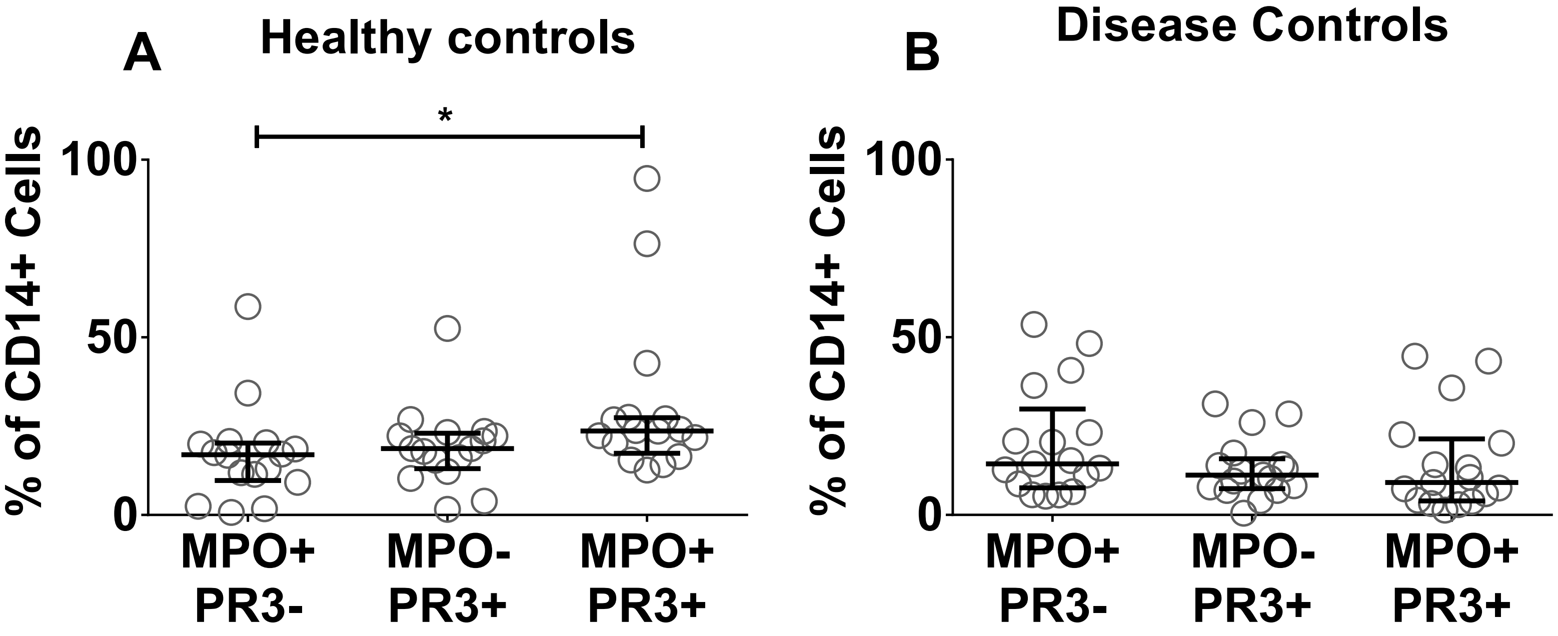
A
Healthy controls
*
% of CD14+ Cells
100
50
0
MPO+ PR3-
MPO- PR3+
MPO+ PR3+
B
Disease Controls
% of CD14+ Cells
100
50
0
MPO+ PR3-
MPO- PR3+
MPO+ PR3+

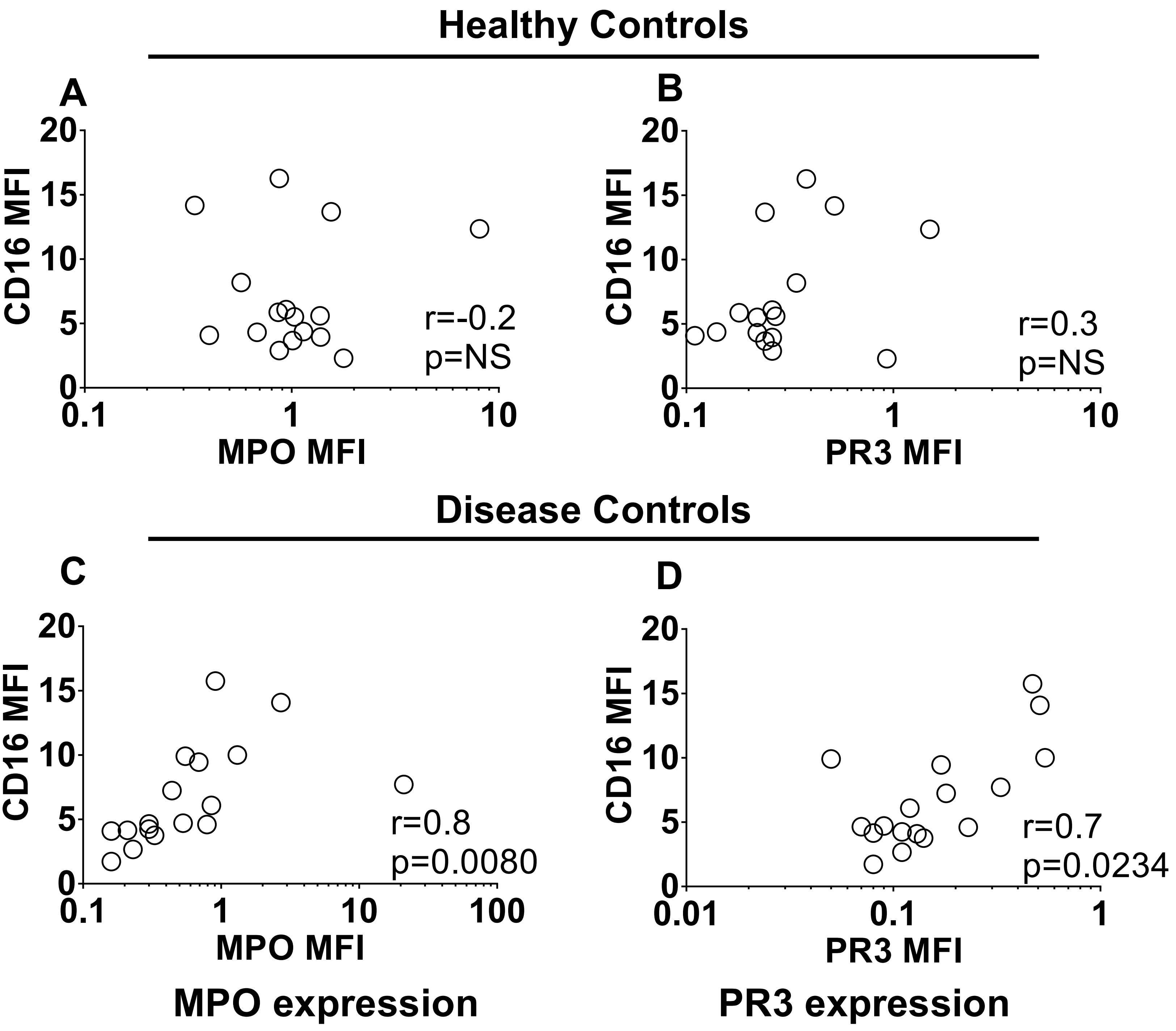
Healthy Controls
A
B
CD16 MFI
20
15
10
5
0
0.1
1
10
MPO MFI
r=-0.2
p=NS
PR3 MFI
r=0.3
p=NS
Disease Controls
C
D
100
r=0.8
p=0.0080
0.01
r=0.7
p=0.0234
MPO expression
PR3 expression

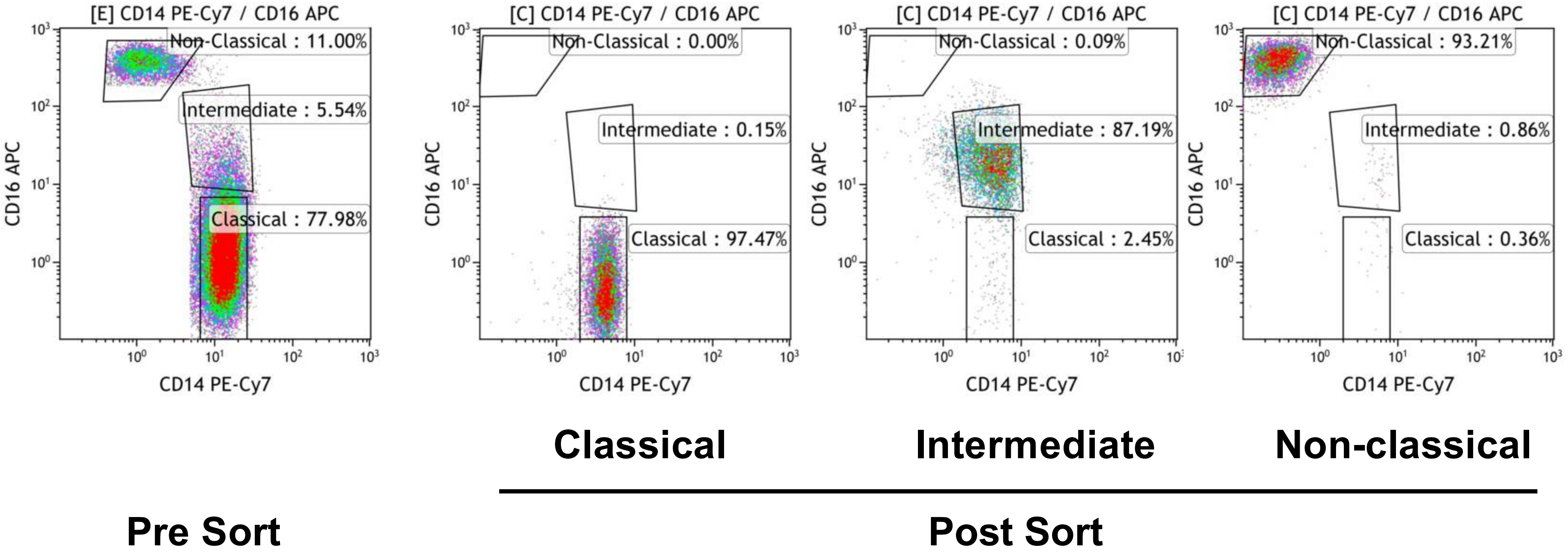
[E] CD14 PE-Cy7 / CD16 APC
Non-Classical : 11.00%
Intermediate : 5.54%
Classical : 77.98%
CD16 APC
CD14 PE-Cy7
[C] CD14 PE-Cy7 / CD16 APC
Non-Classical : 0.00%
Intermediate : 0.15%
Classical : 97.47%
CD16 APC
CD14 PE-Cy7
[C] CD14 PE-Cy7 / CD16 APC
Non-Classical : 0.09%
Intermediate : 87.19%
Classical : 2.45%
CD16 APC
CD14 PE-Cy7
[C] CD14 PE-Cy7 / CD16 APC
Non-Classical : 93.21%
Intermediate : 0.86%
Classical : 0.36%
CD16 APC
CD14 PE-Cy7
Classical
Intermediate
Non-classical
Pre Sort
Post Sort

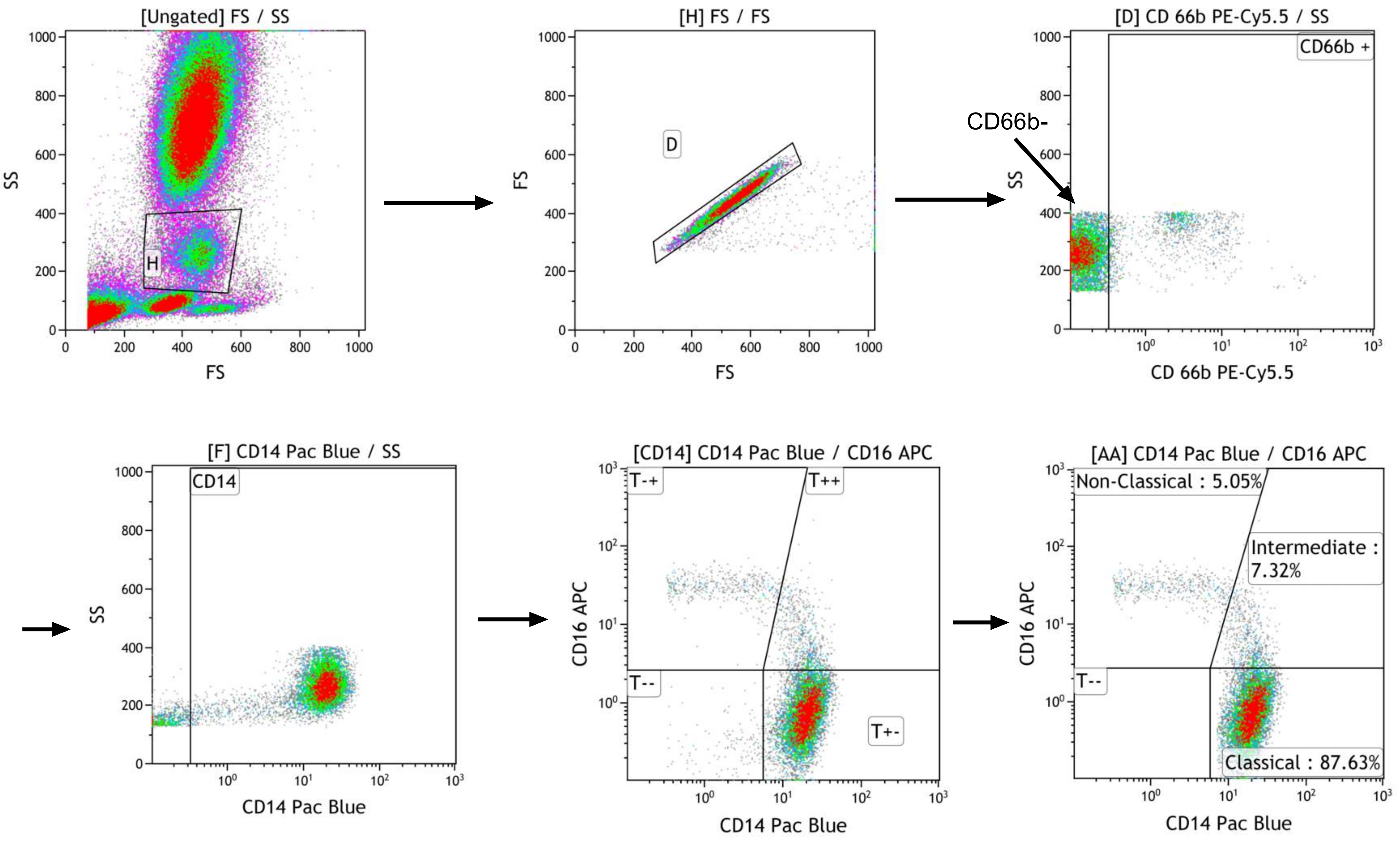
[Ungated] FS / SS
H
[H] FS / FS
D
[D] CD 66b PE-Cy5.5 / SS
CD66b +
CD66b-
[F] CD14 Pac Blue / SS
CD14
[CD14] CD14 Pac Blue / CD16 APC
T-+
T++
T--
T+-
[AA] CD14 Pac Blue / CD16 APC
Non-Classical : 5.05%
Intermediate : 7.32%
T--
Classical : 87.63%
FS
SS
CD 66b PE-Cy5.5
CD14 Pac Blue
CD16 APC

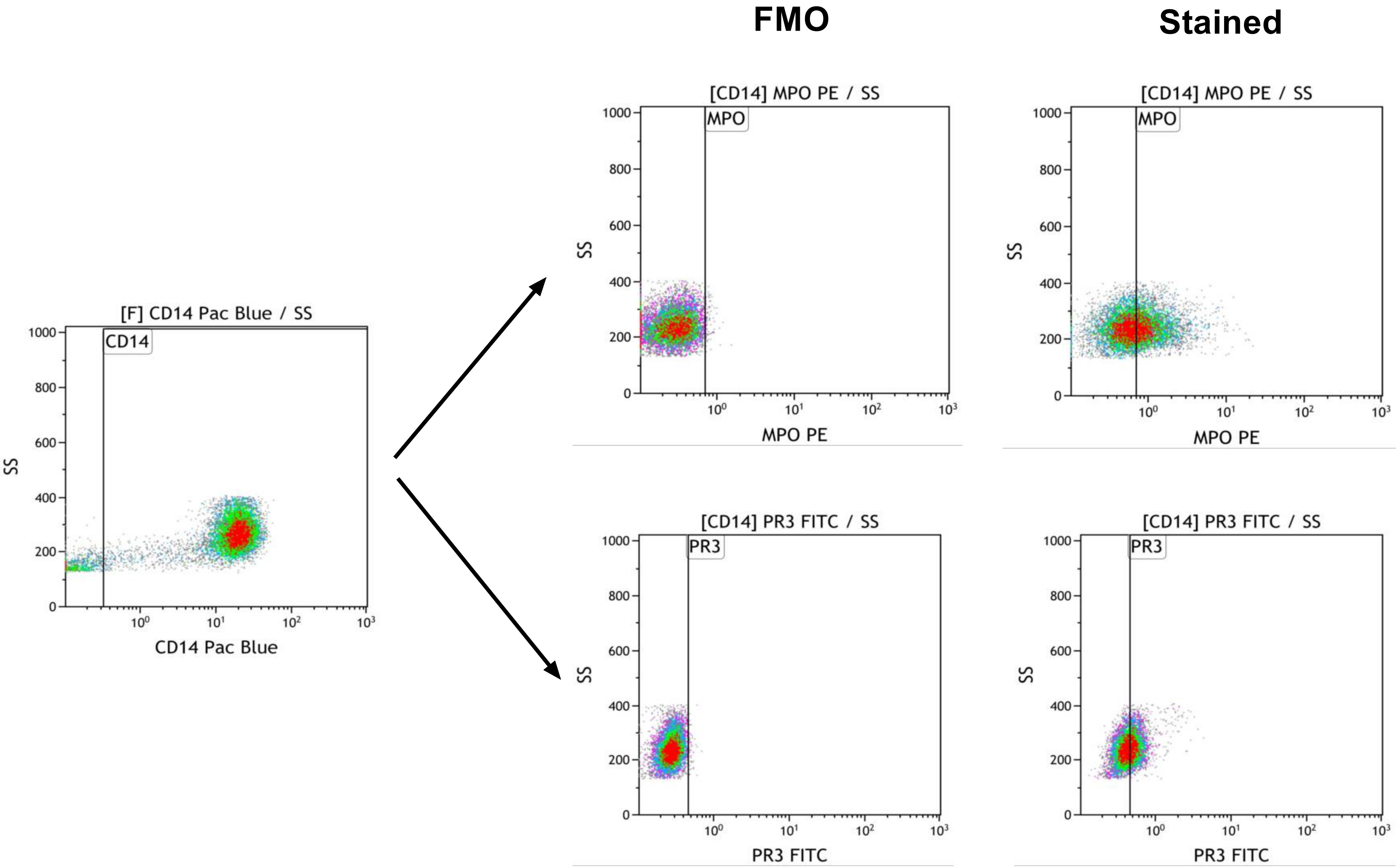

FMO
Stained
[F] CD14 Pac Blue / SS
CD14
CD14 Pac Blue
SS
[CD14] MPO PE / SS
MPO
MPO PE
[CD14] PR3 FITC / SS
PR3
PR3 FITC

Supplemental Figure S1: The ANCA autoantigens MPO and PR3 are preferentially expressed on intermediate monocytes in individuals without vasculitis.

Peripheral blood was collected from healthy and disease control individuals and the percentage of cells expressing cell-surface MPO and PR3 was examined by flow cytometry. The percentage of MPO and PR3 positive cells in each subset is shown for (A - B) healthy controls and (C - D) disease controls. Each symbol represents one individual. Data are presented as the median and interquartile range. Non-parametric one-way ANOVA (Friedman test) and Dunn’s post-test were used to establish significance (*p<0.05, **p<0.01; ***p<0.001; ****p<0.0001). Class: classical; Int: intermediate: NC: non-classical.

Supplemental Figure S2: The MFI of MPO and PR3 expression is increased on intermediate monocytes in control individuals.

Peripheral blood was collected from healthy and disease control individuals. Cells were stained as described in methods and analysed by flow cytometry. Following gating on MPO or PR3 positive cells in each subset, the median fluorescence intensity (MFI) of MPO and PR3 was determined. For each individual these values were corrected by subtraction of the MFI for the fluorescence minus one (FMO) control and expressed relative to the corrected MFI for classical monocytes in that individual. Relative expression of cell-surface MPO or PR3 in each monocyte subset is shown for (A - B) anti-MPO+ AAV patients and (C - D) anti-PR3+ AAV patients. Each symbol represents a separate patient/control individual. Data are presented as the median and interquartile range. Non-parametric one-way ANOVA (Friedman test) and Dunn’s post-test were used to establish significance (*p<0.05, **p<0.01; ***p<0.001). Class: classical; Int: intermediate: NC: non-classical.

Supplemental Figure S3: Cell-surface expression of MPO and PR3 are not linked on monocytes from control individuals.

Peripheral blood was collected from healthy and disease control individuals and the percentage of cells expressing cell-surface MPO and PR3 was examined by flow cytometry. Cells were classified as being MPO+PR3-, MPO-PR3+, MPO+PR3+ or negative for both antigens. Data are presented for (A) healthy controls and (B) disease controls. Each symbol represents an individual. Data are presented as the median and interquartile range. Non-parametric one-way ANOVA (Friedman test) and Dunn's post-test were used to establish significance (* $p<0.05$).

Supplemental Figure S4: Correlation of CD16 expression with MPO and PR3 expression on intermediate monocytes from control individuals.

Peripheral blood was collected from healthy and disease control individuals. Following gating on intermediate monocytes the MFI of CD16 was plotted against that of MPO or PR3. Data presented show (A - B) healthy controls and (C - D) disease controls. Each symbol represents an individual patient. Correlation was tested by Spearman Rank Test.

Supplemental Figure S5: Gating of monocyte subsets.

Monocytes were initially gated based on size (FS) and granularity (SS). Doublets were excluded. Granulocytes were excluded based on positive CD66b staining. CD14+CD66b- monocytes were subdivided into classical, intermediate and non-classical subsets based on CD14 and CD16 staining. Flow plots represent a typical AAV patient.

Supplemental Figure S6: of monocyte surface MPO and PR3 expression

Initially monocytes were gated as shown in Supplemental Figure S5. MPO and PR3 positive cells were subsequently defined using fluorescence minus one (FMO) controls. Flow plots represent a typical example.

Supplemental Figure S7: Purity of monocyte subsets following sorting

Monocytes were gated based on size (FS) and granularity (SS). Doublets were excluded. Purity of sorted subsets was found by subdividing CD14+ monocytes into classical, intermediate and non-classical subsets based on CD14 and CD16 staining. Flow plots show typical pre- and post-sort monocyte subsets proportions.
